# Development and application of an updated haplotype reference panel for association analysis of spontaneous sex reversal in XX rainbow trout

**DOI:** 10.3389/fgene.2025.1692544

**Published:** 2025-12-10

**Authors:** Sixin Liu, Gregory M. Weber, Kyle E. Martin, Roseanna Long, Jill E. Birkett, Yniv Palti

**Affiliations:** 1 National Center for Cool and Cold Water Aquaculture, Agricultural Research Service, United States Department of Agriculture, Kearneysville, WV, United States; 2 Troutlodge Inc., Sumner, WA, United States

**Keywords:** rainbow trout, low-coverage whole-genome sequencing, genotype imputation, haplotype reference panel, SNP, low-pass sequencing, spontaneous male, sex reversal

## Abstract

With the rapid cost reduction of next-generation sequencing, low-coverage whole-genome sequencing (lcWGS) followed by genotype imputation is becoming a cost-effective alternative to SNP (single nucleotide polymorphism) array genotyping. Previously, we constructed a reference panel consisting of 410 samples representing five breeding populations of rainbow trout (*Oncorhynchus mykiss*). However, the reference panel had a limited representation of the major commercial populations in the U.S. The objectives of this study were two-fold: 1) to update the haplotype reference panel of rainbow trout by adding more reference populations and more samples from the previous reference populations; and 2) to identify SNPs associated with spontaneous sex reversal to males in XX rainbow trout (sXX sex reversal). To update the reference panel, high-coverage whole-genome sequences were obtained from 129 additional fish from several populations. To identify SNPs associated with sXX sex reversal, samples from two families were genotyped with both the Axiom 57K SNP array and lcWGS. The updated reference panel outperformed the previous panel with an increase in accuracy of genotype imputation and a reduction in low-confidence genotypes. Based on the array genotypes, 55 significant SNPs associated with sXX sex reversal were identified and 53 out of the 55 SNPs were located on chromosome OmyA26. Based on the imputed genotypes, 743 SNPs on chromosome OmyA26 and 7 SNPs on chromosome OmyA19 were associated with sXX sex reversal. Two of those OmyA26 significant SNPs were identified by both genotyping methods. In conclusion, the updated haplotype reference panel improved the accuracy of genotype imputation from lcWGS, and enabled identification of additional SNPs associated with sXX sex reversal in rainbow trout.

## Introduction

Low-coverage whole-genome sequencing (lcWGS) followed by genotype imputation has emerged as an alternative cost-effective approach for genome-wide high-density genotyping (Davies et al., 2021; Rubinacci et al., 2023). The application of this emerging genome-wide genotyping method has been widely documented in many recent genetic studies in humans (Flanagan et al., 2024; Herzig et al., 2024), terrestrial livestock such as pig (Wang YX. et al., 2025) and sheep (Li et al., 2025), and aquaculture species such as salmon (Gundappa et al., 2025) and scallop (Wang YJ. et al., 2025). Previously, we developed and reported a haplotype reference panel of rainbow trout (*Oncorhynchus mykiss*), and the reference panel was used for accurate genotype imputation in two breeding populations (Liu et al., 2024). However, this reference panel had limited representation of the major commercial aquaculture populations in the U.S. Among the four populations marketed by the largest rainbow trout egg distributor in the U.S., Troutlodge Inc., the February spawning population was not included, and the August spawning population was represented by only 19 samples. Thus, it is necessary to improve the reference panel by adding more reference populations and more samples from the previous reference populations.

Rainbow trout is one of the most widely cultured cold freshwater fish with a global production of about 953,000 tons in 2021 (FAO, 2024). All-female populations are preferred and widely used for rainbow trout production to avoid the production losses and reductions in product quality associated with early sexual maturation in males. Although rainbow trout are gonochoristic and have a male heterogametic (males XY, females XX) sex determination system controlled by a major sex-determining gene, *sdY* (Yano et al., 2012), spontaneous males were reported in genetically all-female XX populations including gynogenetic offspring (Quillet et al., 2002), commercial farm populations (Fraslin et al., 2020) and experimental families (Weber et al., 2023). Based on genome-wide association studies, four QTLs (Quantitative Trait Loci) for spontaneous sex reversal to males in XX rainbow trout (sXX sex reversal) were identified in a commercial farm population in France (Fraslin et al., 2020). Recently, these four QTLs were validated in additional commercial rainbow trout populations from France (Dehaullon et al., 2025). Nevertheless, four different QTL for sXX sex reversal were identified in another strain of rainbow trout from France (Guyomard et al., 2014). After crossing females from an American commercial strain spawned in August with cryopreserved milt from homogametic XX neomales of an American commercial February strain, 30 spontaneous males were unexpectedly observed among 344 progenies at about 16 months post-hatch (Weber et al., 2023). Another generation of offspring was obtained for genetic studies by crossing the spontaneous XX males with ovulating females. The objectives of this study were two-fold: 1) to update the haplotype reference panel of rainbow trout by adding more reference populations and more samples from the previous reference populations; and 2) to identify SNPs associated with sXX sex reversal in rainbow trout.

## Materials and methods

### Additional reference samples of rainbow trout

A total of 129 additional reference samples (Table 1; Supplementary Table S1) were used to update the reference panel. We added 43 fish from the Troutlodge August population (TLUA) and 5 fish from the Troutlodge May population (TLUM). Three new reference populations were also added to the reference panel, including 66 fish from the Troutlodge February population (TLUF), four fish from the breeding population at USDA-ARS Hagerman Fish Culture Experiment Station (HFCES), and 11 fish derived from the hybrid crosses between the TLUA and TLUF populations (TLUA/F).

**TABLE 1:** Reference samples used to develop an updated haplotype reference panel for genotype imputation in rainbow trout*.

Populations	Spawning month	Number of samples		
		Previous	Additional	Total
NCCCWA even-year	February	84	0	84
NCCCWA odd-year	February	84	0	84
TLUF	February	0	66	66
TLUM	May	76	5	81
TLUA	August	19	43	62
HFCES	August	0	4	4
TLUA/F	November	0	11	11
TLUN	November	147	0	147
Total	​	410	129	539

*NCCCWA, national center for cool and cold water aquaculture; TLUF, Troutlodge February spawning population; TLUM, Troutlodge May spawning population; TLUA, Troutlodge August spawning population; TLUN, Troutlodge November spawning population; TLUA/F, the hybrids between TLUA, and TLUF; HFCES, hagerman fish culture experiment station.

### Two QTL mapping families for association analysis of sXX sex reversal

The spontaneous XX males, identified from genetically all XX female rainbow trout in the experimental families from the hybrid crosses between TLUA females and cryopreserved milt of TLUF neomales (Weber et al., 2023), provided an opportunity to identify SNP markers associated with sXX sex reversal. Since the sample sizes were small for the original families, another generation of random crosses were made between spontaneous XX males and ovulating females derived from the hybrid crosses. Two full-sib families from this second generation, ST15 and ST22, were used in this study because high proportions of spontaneous XX males (Table 2) were observed in these two families. The fish were raised under the standard culture protocol at the National Center for Cool and Cold Water Aquaculture, as described in detail in Weber et al. (2023). At about 12 months post-hatch, 464 fish from family ST15 and 57 fish from family ST22 were euthanized with 250 mg/L of Tricaine-S, and gonads were examined for sex classification. Fish with one or two testes without observable ovarian tissue were recorded as male. Fish with one or two ovaries without observable testicular tissue were recorded as female. Fish with both testicular and ovarian tissues were recorded as intersex. Gonads that were too small to be classified by unaided visual examination were further examined using a binocular dissecting microscope. These fish were recorded as immature male, immature female or immature intersex using the same criteria described above. Additional mature males were identified in these two families from fish saved as breeding candidates. We focused on the binary phenotypes, male and female, for association analyses in this study, and the samples selected for genotyping are summarized in Table 2. Among the 285 offspring used for SNP array genotyping, 261 samples were also sequenced with a target of 0.5x genome coverage (Table 2). The four parents were also included for array genotyping and high-coverage whole-genome sequencing with a target of 30x genome coverage.

**TABLE 2:** Number of offspring genotyped with SNP array and low-coverage whole-genome sequencing*.

Family	SNP array		Low-coverage sequencing	
	Males	Females	Males	Females
ST15	91 (88)	99 (96)	91 (89)	99 (98)
ST22	60 (50)	35 (30)	59 (59)	12 (12)
Total	151 (138)	134 (126)	150 (148)	111 (110)

*The number of samples retained for association analysis after quality filtering are listed within parentheses.

### DNA sequencing and read mapping

Fin clips were preserved in 95% ethanol until DNA extractions, and DNA was extracted from fin clips following the manufacturer recommended protocols for AutoGenprep 965 (Autogen, Holliston, MA, USA). The DNA samples were sequenced in paired-end (2 × 150 bp) mode on Illumina sequencers. The 129 additional reference samples were sequenced with a target of 30x genome coverage, and selected samples (Table 2) of the two QTL mapping families were sequenced with a target of 0.5x genome coverage. The raw sequence reads were trimmed with trimmomatic v0.38 (Bolger et al., 2014) to remove adapter sequences and low-quality bases. Quality-trimmed reads were mapped to the rainbow trout reference genome USDA_OmykA_1.1 (Gao et al., 2021) using the BWA-mem2 v2.2.1 (Vasimuddin et al., 2019). We used a prefix OmyA to name each chromosome because the genome of Arlee was used as the reference genome. Duplicated reads were marked with the MarkDuplicates tool of GATK v4.3 (Van der Auwera and O'Connor, 2020) after the sequence alignments were sorted by coordinates using SAMtools v1.16.1 (Li et al., 2009). The overall genome coverage for each sample was calculated using mosdepth v0.3.3 (Pedersen and Quinlan, 2018).

### Variant calling, filtering and SNP analyses

We combined the 129 additional reference samples with the 410 reference samples reported in our previous study (Liu et al., 2024) to call genetic variants using GATK v4.3. Briefly, HaplotypeCaller was used to call genetic variants per sample to produce a file in GVCF format. Then, GenomicsDBImport was used to consolidate all GVCF files across all samples into a database, and GenotypeGVFs was used for joint genotyping to call SNPs and small insertion/deletion variants. VariantFiltration was used to filter out low quality SNPs with the following criteria: ExcessHet > 54.69, QD < 2.0, QUAL < 30.0, SOR > 3.0, FS > 60.0, MQ < 40.0, MQRankSum < −12.5 or ReadPosRankSum < −8.0. Only biallelic SNPs were retained using BCFtools v1.16 (Danecek et al., 2021), and the SNPs were further filtered with VCFtools v0.1.16 (Danecek et al., 2011) using the following arguments: -minGQ 10 --minDP 4 --max-meanDP 28 --max-missing 0.9 --maf 0.005. SnpEff v5.1d (Cingolani et al., 2012) was used to annotate the SNPs, and PLINK v1.19 (Chang et al., 2015) was used for principal component analysis with 150,000 random SNPs as described in our previous study (Liu et al., 2024).

### SNP array genotyping

A total of 285 offspring (Table 2) and four parents of the two families were genotyped with the 57K rainbow trout genotyping array (Palti et al., 2015) following manufacturer’s instructions. To improve the accuracy of genotype calls, the 289 samples were combined with 2,300 TLUF samples genotyped previously to call genotypes following the user guide for Axiom genotyping array data analysis, and samples with a call rate lower than 95% were removed from further analysis. Initially, we extracted only the genotypes of samples from family ST15, and set genotypes with Mendelian errors to missing genotypes using the option--set-me-missing of PLINK v1.9. Monomorphic SNPs and SNPs with missing data greater than 10% were filtered out. Then, we further filtered out the SNPs with extreme genotype ratios. There are three possible genotypes for each SNP, A1A1, A1A2 and A2A2, where A1 stands for the minor allele and A2 stands for the major allele. For SNPs that were heterozygous for both parents, we retained only SNPs with genotype counts greater than 30 for each of the three genotype groups. For SNPs that were heterozygous for only one of the two parents, we retained the SNPs if the genotype counts were greater than 40 for both A1A2 and A2A2, and the A1A1 counts were less than 12. After data filtering, 22,228 SNPs were retained in family ST15.

Genotypes of family ST22 were extracted for the 22,228 SNPs retained in family ST15. SNPs with extreme genotype ratios were identified using the same method described above except that the thresholds were reduced to half due to a smaller sample size of family ST22. To exclude SNPs with extreme genotype ratios from association analyses, we set the genotypes of the two parents of family ST22 to missing genotypes for those SNPs. Finally, we merged the quality filtered genotype files of families ST15 and ST22 for association analyses, and the merged genotype data were deposited to Ag Data Commons (DOI: 10.15482/USDA.ADC/30670556).

### Phasing and genotype imputation

The SNP genotypes of the 539 reference samples were phased using SHAPEIT5 v5.1.1 (Hofmeister et al., 2023), and the haplotypes were used as references to impute the genotypes of families ST15 and ST22 from lcWGS using GLIMPSE2 (Rubinacci et al., 2023). To compare the accurary of genotype imputation with different reference panels, we also imputed the genotypes of families ST15 and ST22 using our previous haplotype reference panel (Liu et al., 2024). To remove low-confidence genotypes, genotypes with a posterior probability less than 0.90 were set to missing genotypes using BCFtools v1.16.

We compared the imputed genotypes with the genotypes based on SNP array. Like our previous study (Liu et al., 2024), three metrics, concordance, squared Pearson correlation coefficient (*r* ^2^) and nonreference discordance (NRD), were used to measure the accuracy of genotype imputation from lcWGS data. Concordance refers to the percentage of identical genotypes between imputed genotypes and array genotypes, and *r* ^2^ was calculated from the alternative allele dosages of imputed genotypes and array genotypes. NRD = 100 x (*e*_rr _+ *e*_ra _+ *e*_aa_)/(*e*_rr _+ *e*_ra _+ *e*_aa _+ *m*_ra _+ *m*_aa_), where *e*_rr_, *e*_ra_ and *e*_aa_ are counts of the mismatches for the homozygous reference, heterozygous and homozygous alternative genotypes, respectively, and *m*_ra_ and *m*_aa_ are the counts of the matches at the heterozygous and homozygous alternative genotypes. All three metrics were calculated using the stats command of BCFtools v1.16.

### Association analyses of sXX sex reversal

Before performing association analyses, we filtered the imputed genotypes of the two families, ST15 and ST22. First, the genotypes of the four parents of the two families were extracted from the reference panel. We then extracted the imputed genotypes of family ST15 and retained only polymorphic SNPs. Using the same criteria used to filter the array genotypes described above, SNPs with extreme genotype ratios were also filtered out from the imputed genotypes. A total of 4,978,369 polymorphic SNPs were retained after genotype quality filtering. We then extracted the imputed genotypes of family ST22 for the SNPs retained in family ST15. We also searched for SNPs with extreme genotype ratios in the family ST22 using the same criteria described above to filter the array genotypes. Then, we set the genotypes of the two parents of family ST22 to missing genotypes for SNPs with extreme genotype ratios. Finally, we merged the filtered imputed genotypes of the two families. Three samples were excluded from association analysis because they had 15% or more missing genotypes.

The Transmission Disequilibrium Test (TDT) is a statistical approach for family-based association analysis (Spielman et al., 1993). TDT is based on the transmission of marker alleles from parents to offspring to detect linkage-disequilibrium between markers and a binary trait of interest. PLINK v1.9 was used for TDTs to identify SNPs associated with sXX sex reversal in this study. The males were assigned a phenotype value of 2, and the females were assigned a phenotype value of 1. SNPs with MAF <0.15 were filtered out from the TDT results. The significant thresholds were determined with a conservative Bonferroni correction. For the array genotypes, SNPs with p-value less than 2.25e-6 (0.05/22,228) were considered significant. For the imputed genotypes, SNPs with p-value less than 1e-8 (0.05/4,978,369) were considered significant.

## Results

### Development of an updated haplotype reference panel

**FIGURE 1:**
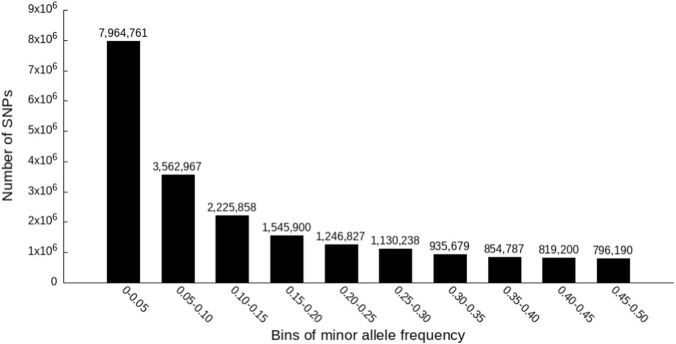
Distribution of SNPs by bins of minor allele frequency.

A total of 129 additional reference samples were sequenced in this study. The depths of genome coverage ranged from 17x to 49.5x with an average of 26.3x (Supplementary Table S1). SNPs were called after combining the sequence data of the 129 samples with the 410 reference samples reported in our previous study (Liu et al., 2024). After quality filtering, 21,082,407 biallelic SNPs were retained. Of those retained SNPs, 7,964,761 (37.8%) SNPs had MAFs of 0.05 or less, and 13,117,646 (62.2%) SNPs had MAFs greater than 0.05 (Figure 1). The ratio of transitions to transversions was 1.03. SNP annotation using SnpEff revealed 9,232 SNPs with high impact, and 544,006 SNPs with moderate impact on gene functions. Also, 60.8% of the SNPs are in introns (Supplementary Figure S1).

**FIGURE 2:**
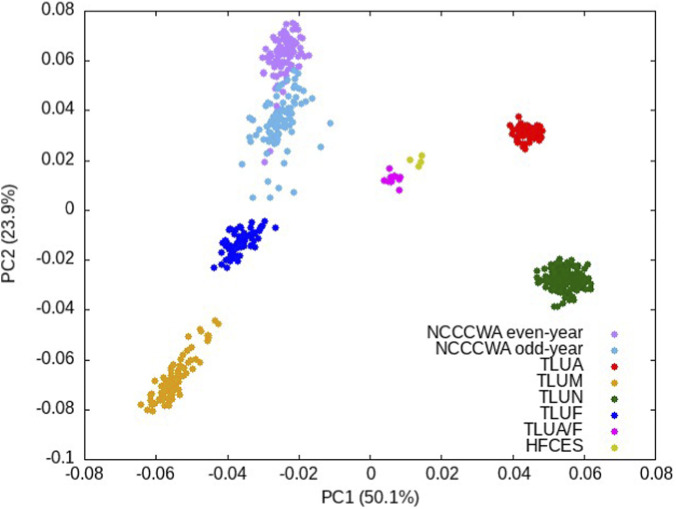
Population structure of the 539 reference samples based on the top two principal components. The samples were color-coded as shown in the legend.

Principal component analysis was used to identify population structure among the 539 reference samples (Figure 2). The first principal component explained 50.1% of the population stratification variance and separated the samples by spawning dates, and the second principal component explained 23.9% of the population stratification variance and separated the three February spawning populations, TLUF and two NCCCWA populations. This result is similar to the population structure that we reported recently (Liu et al., 2024), and is consistent with our previous studies of the population structure of North America farmed rainbow trout (Liu et al., 2021; Liu et al., 2017).

### Evaluation of the accuracy of genotype imputation

We used both the updated haplotype reference panel described above and the haplotype reference panel that we reported previously (Liu et al., 2024) to impute genotypes of the samples sequenced to about 0.5x genome coverage. We then compared the imputed genotypes with the genotypes based on SNP array to evaluate the accuracy of genotype imputation using three metrics, concordance, *r* ^2^ and NRD. Surprisingly, both panels performed very well with highly accurate imputed genotypes (Table 3). But, the updated haplotype reference panel outperformed slightly the previous reference panel for all three metrics (Table 3). Furthermore, the updated reference panel again outperformed the previous panel with a higher number of retained SNPs and a lower rate of missing genotypes (Table 4).

**TABLE 3:** Accuracy of genotype imputation from low-coverage whole-genome sequencing data using the updated and previous haplotype reference panels*.

References panel	Concordance (%)	NRD (%)	*r* ^2^
Updated	98.4	2.4	0.97
Previous	97.9	3.1	0.96

*NRD, nonreference discordance; *r* ^2^, squared Pearson correlation coefficient.

**TABLE 4:** Quality of genotypes imputed from low-coverage whole-genome sequencing data using the updated and previous haplotype reference panels.

References panel	No. of SNPs	SNPs with missing genotypes >10%	Missing genotypes
Updated	21,082,407	2,080,780	4.0%
Previous	20,434,612	3,298,439	5.1%
Updated - Previous	647,795	−1,217,659	−1.1%

### Identification of SNPs associated with sXX sex reversal

High proportions of spontaneous males were observed in both families, ST15 and ST22. Among the 464 fish dissected for sex phenotypes in family ST15, 129 (27.8%) males and 14 (3.0%) intersex fish were recorded (Table 5). Among the 57 fish dissected for sex phenotypes in family ST22, 43 (75.4%) males and 1 (1.8%) intersex fish were observed (Table 5). To increase the number of samples used for array genotyping and lcWGS, the fish saved as breeding candidates were checked for spermiating males. Among the 20 breeding candidates from family ST15, 10 spermiating males were identified. For family ST22, 25 out of 48 breeding candidates were spermiating males.

**TABLE 5:** Sex phenotypes observed in families ST15 and ST22.

Families	ST15		ST22	
	Count	Percentage	Count	Percentage
Male	129	27.8%	43	75.4%
Intersex	14	3.0%	1	1.8%
Female	321	69.2%	13	22.8%
Total	464	100.0%	57	100.0%

Using the combined array genotypes of the two families, 55 SNPs associated with sXX sex reversal were identified (Table 6; Supplementary Table S2). Of those 55 significant SNPs, 53 SNPs were located on chromosome OmyA26, one was on chromosome OmyA3, and one was on chromosome OmyA17. With the genotypes imputed from lcWGS, 743 SNPs located on chromosome OmyA26 were significantly associated with sXX sex reversal (Table 6; Supplementary Table S3). Two of those significant OmyA26 SNPs were identified by both genotyping methods. Unexpectedly, there were no significant SNPs on chromosome OmyA3 and OmyA17, and seven SNPs on chromosome OmyA19 were significantly associated with sXX sex reversal based on association analyses using the imputed genotypes (Table 6; Supplementary Table S3).

**TABLE 6:** Number of significant SNPs associated with spontaneous sex reversal in XX rainbow trout using two genome-wide genotyping approaches.

Chromosomes	Array genotypes	Imputed genotypes	Shared SNPs
OmyA3	1	0	0
OmyA17	1	0	0
OmyA19	0	7	0
OmyA26	53	743	2

## Discussion

### An updated haplotype reference panel

Previously, we developed a haplotype reference panel of rainbow trout, and the reference panel was used for accurate genotype imputation in two breeding populations (Liu et al., 2024). In this study, we obtained high-coverage whole-genome sequences for 129 additional reference samples and updated the haplotype reference panel. The updated panel included samples from additional reference populations and increased the sample sizes of the reference populations included in the previous panel. There were 647,795 more SNPs in the updated panel than in our previous reference panel. This result is consistent with results reported in other species. Increased sample sizes and genetic diversity led to more SNPs being identified by large-scale whole-genome sequencing projects in various species such as humans (Bick et al., 2024), cattle (Hayes and Daetwyler, 2019) and pig (Du et al., 2024).

Although many factors such as the composition and size of the haplotype reference panel affect the accuracy of genotype imputation (Ding et al., 2023; Lloret-Villas et al., 2023; Rubinacci et al., 2023), the relatedness between reference samples and target samples has a major impact on the accuracy of genotype imputation (Martin et al., 2021). The target samples used for lcWGS in this study were derived from the hybrid crosses between the TLUA and TLUF breeding populations. Only 19 TLUA samples and none from the TLUF population were included in the previous reference panel. For the updated reference panel, we added 66 TLUF samples, 43 TLUA samples, and 11 samples derived from the hybrid crosses between the TLUA and TLUF breeding populations. Thus, it makes sense that the updated reference panel outperformed the previous panel with an increase in accuracy of genotype imputation and a reduction in low-confidence genotypes.

### SNPs associated with sXX sex reversal in rainbow trout

Most of the SNPs associated with sXX sex reversal reported in this study were located on chromosome OmyA26. This result is consistent with the results reported by Guyomard et al. (2014). In that study, two double haploid families were used to map QTLs for sXX sex reversal in rainbow trout, and four QTLs were identified. One of the four QTL was significant at the genome-wide level, and this QTL was located on chromosome OmyA26 (Guyomard et al., 2014). However, Guyomard et al. (2014) did not report the sequences containing the SNPs associated with the OmyA26 QTL, it was not possible to test whether the OmyA26 QTL identified in both studies were co-located in the same region of the chromosome. For the OmyA26 QTL reported in this study, the SNP alleles increasing the risk of sXX sex reversal were derived from the sires. Reduced genetic recombination in males has been well documented in rainbow trout (Pearse et al., 2019). Furthermore, high linkage disequilibrium was observed in rainbow trout aquaculture breeding populations (Vallejo et al., 2018). Low recombination rates in males and high linkage disequilibrium are the likely reasons why so many SNPs from a large region of 15 Mb on chromosome OmyA26 were significantly associated with sXX sex reversal in this study. We will perform fine mapping and identify candidate genes in the future.

SNPs located on other chromosomes were also associated with sXX sex reversal in this study. Using the SNP array genotypes for association analyses, one SNP on chromosome OmyA3 and one on OmyA17 were significantly associated with sXX sex reversal. However, association analyses using the imputed genotypes from lcWGS did not uncover any significant SNPs on those two chromosomes. Therefore, those two significant SNPs were likely due to false positive results. Seven significant SNPs on chromosome OmyA19 were identified using the imputed genotypes from lcWGS. None of those 7 SNPs is on the SNP array, which might explain why the association analyses with the SNP array genotypes did not reveal any significant SNPs on chromosome OmyA19. Thus, it would be interesting to evaluate in the future whether the high-density imputed genotypes can contribute to the identification of additional regions associated with traits of interest in rainbow trout, which have not been detected with array or other low-density genotyping methods.

SNPs associated with sXX sex reversal were identified and validated in commercial rainbow trout populations from France (Dehaullon et al., 2025; Fraslin et al., 2020), and they were located on chromosomes Omy1, Omy12 and Omy20. However, we did not identify any significant SNPs located on those three chromosomes in the current study. The most likely explanation for the discrepancy is that SNPs associated with sXX sex reversal might be population or even family specific. Consistent with this explanation, not all QTLs for sXX sex reversal reported by Fraslin et al. (2020) were validated in all six populations used for QTL validation (Dehaullon et al., 2025). This can also explain why different QTLs for sXX sex reversal were detected from two double haploid mapping populations of rainbow trout (Guyomard et al., 2014). The samples used for association analyses in this study were derived from North American commercial rainbow trout populations, and the rates of spontaneous males were high. The rainbow trout populations from France used for the initial association mapping had only 1.4% spontaneous males (Fraslin et al., 2020). In contrast, each of the two families used in this study had 27.8% or more spontaneous males. Nonetheless, we acknowledge that false positive or false negative association results reported in this study could be another reason for the discrepancy.

All-female lines are preferred for rainbow trout production because early sexual maturation in males causes reductions in production efficiency and product quality. Thus, it is desirable to avoid spontaneous males in the populations used for rainbow trout aquaculture production. In this study, we identified SNPs associated with sXX sex reversal using two QTL mapping families. However, it is necessary to validate the results before using these SNPs to select against spontaneous males. Additional crosses have been made and will be used to validate the results of this study using a larger sample size.

### Comparison of the two genome-wide genotyping methods

Both SNP array genotyping and lcWGS followed by genotype imputation were used to genotype the two families used in this study. For the SNP array genotyping, about 22K SNPs were retained after quality and polymorphism filtering. For lcWGS followed by genotype imputation, about 5 million polymorphic SNPs were retained after genotype quality filtering. The large number of imputed genotypes allowed us to identify many more SNPs associated with the OmyA26 QTL, and also enabled us to identify an additional QTL on chromosome OmyA19. The significant improvement of association analysis using imputed genotypes is in line with the results reported in other species. For example, imputed genotypes from lcWGS significantly improved the resolution of genetic mapping in pig (Ding et al., 2023), and a recent sequence-based association analysis revealed additional variants associated with milk production traits in dairy cattle (Krizanac et al., 2025). Therefore, the updated haplotype reference panel reported in this study can facilitate high resolution association analysis in rainbow trout.

## Data Availability

The raw sequence reads generated in this study were deposited in the NCBI Sequence Read Archive under BioProject PRJNA1297171. The SNP array genotype data were deposited to Ag Data Commons (DOI: 10.15482/USDA.ADC/30670556).

## Supplementary material

SUPPLEMENTARY TABLE S1 Detailed information about the 129 reference samples.

SUPPLEMENTARY TABLE S2 Significant SNPs associated with sXX sex reversal based on association analyses using SNP array genotypes.

SUPPLEMENTARY TABLE S3 Significant SNPs associated with sXX sex reversal based on association analyses using imputed genotypes from lcWGS.

SUPPLEMENTARY FIGURE S1 Distribution of SNPs by genomic regions.
