# Supplementary figures and images for: Development and application of an updated haplotype reference panel for association analysis of spontaneous sex reversal in XX rainbow trout
Source: Front Genet. 2025 Dec 10;16:1692544. doi: 10.3389/fgene.2025.1692544 (PMC12726600; doi:10.3389/fgene.2025.1692544)

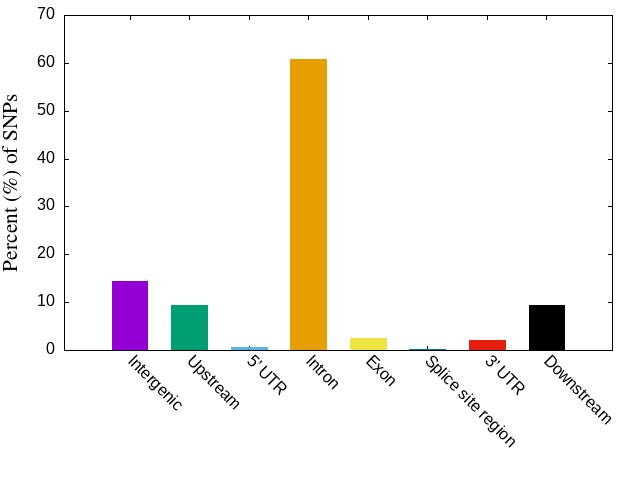

Supplement: Supplementary file 1 [file Image1.jpeg]
